# The role of machine learning methods in physiological explorations of endurance trained athletes: a mini-review

**DOI:** 10.3389/fspor.2024.1440652

**Published:** 2024-11-21

**Authors:** Félix Boudry, Fabienne Durand, Henri Meric, Amira Mouakher

**Affiliations:** ^1^ Espace Dev, Université de Perpignan Via Domitia, Perpignan, France; ^2^ UMR Espace Dev (228), Université Montpellier, IRD, Montpellier, France

**Keywords:** endurance trained athletes, exploration, machine learning (ML), exercise physiology, performance

## Abstract

Endurance-trained athletes require physiological explorations that have evolved throughout the history of exercise physiology with technological advances. From the use of the Douglas bag to measure gas exchange to the development of wearable connected devices, advances in physiological explorations have enabled us to move from the classic but still widely used cardiopulmonary exercise test (CPET) to the collection of data under real conditions on outdoor endurance or ultra-endurance events. However, such explorations are often costly, time-consuming, and complex, creating a need for efficient analysis methods. Machine Learning (ML) has emerged as a powerful tool in exercise physiology, offering solutions to these challenges. Given that exercise physiologists may be unfamiliar with ML, this mini-review provides a concise overview of its relevance to the field. It introduces key ML methods, highlights their ability to predict important physiological parameters (e.g., heart rate variability and exercise-induced hypoxemia), and discusses their strengths and limitations. Finally, it outlines future directions based on the challenges identified, serving as an initial reference for physiologists exploring the application of ML in endurance exercise.

## Introduction

The rapid advancement in computational power has catalyzed the development of artificial intelligence (AI) techniques, particularly ML. Artificial intelligence involves creating systems capable of tasks that typically require human intelligence, such as learning, reasoning, and decision-making (1). Within this framework, ML, a subset of AI, refers to algorithms that enable computers to learn from data without explicit programming. These algorithms identify patterns and make decisions or predictions by improving their performance over time with experience (2). This potential has led to valuable applications across various fields, including exercise physiology.

Exercise physiology focuses on understanding the body's responses to physical effort. This field has a rich history dating back to 1889 (3), but labor-intensive data collection and analysis have historically constrained its development (4). Modern technologies, such as automated gas analyzers used during cardiopulmonary exercise testing (CPET), have improved efficiency. Often used to assess cardiorespiratory and metabolic responses in endurance athletes, CPET presents several limitations (5). Expansive and bulky equipment is required, and the whole process, including subject preparation, testing, and data analysis, can be time-consuming. Additionally, CPET is often inaccessible to many practitioners and requires qualified personnel. Despite these drawbacks, CPET is valuable for athletes, determining key parameters for endurance performance like maximal oxygen uptake (VO_2_max) and ventilatory thresholds, concepts that can be challenging to interpret (6), or detecting cardiac events. Moreover, exercise-induced hypoxemia (EIH) exhibited by some endurance athletes, leading to specific adaptations to exercise, is a phenomenon measured during CPET based on the fall of pulse oxygen saturation (SpO_2_) (7).

With the development of endurance and ultra-endurance activities, physiologists need to explore physiological parameters in ecological conditions (8, 9). On one hand, the miniaturization of technologies over the last few decades has made it possible to develop portable metabolic systems that can be used in free-living environments (10, 11). However, these systems still share some drawbacks with CPET, such as bulkiness and complexity. On the other hand, advancements in wearable technology have significantly impacted exercise data collection, enabling real-time monitoring of performance parameters (6, 12). Devices like smartwatches and biosensors can continuously track physiological parameters during endurance exercise (13) and generate extensive data, though much of this complex data remains underutilized.

Physiologists confront the analysis, interpretation, and management of big and complex datasets, regardless of the context of physiological data exploration in endurance exercise. Traditional statistical methods often struggle with the complexity of handling nonlinear and multivariate data relationships inherent in physiological data. As machine learning methods continue to evolve, they offer novel ways to process such data, which is essential in fields like exercise physiology. Machine learning algorithms excel at identifying hidden patterns within these datasets, providing new insights into how the body responds to exercise. For instance, ML models have been used to predict key physiological outcomes, such as VO_2_max, based on non-invasive data, offering practical alternatives to traditional testing methods (14). However, the integration of ML in exercise physiology also presents some limitations and challenges.

This mini-review aims to discuss the types of ML methods in exercise physiology and their strengths and limitations, providing a comprehensive overview of the current state of the art regarding the role of ML methods in the field of exercise endurance physiology, with a focus on key physiological parameters for endurance performance.

## Machine learning methodologies

There are numerous and diverse ML methods, each tailored to specific tasks and data types (15). Machine learning methods can be classified into four categories: supervised learning, unsupervised learning, semi-supervised learning, and reinforcement learning.

### Supervised learning

Supervised learning methods involve training models on labeled data. The goal is for the model to generalize to new, unseen data. Like a student learning from a teacher, supervised learning requires supervision during training (16). Common supervised learning algorithms include decision trees, random forests, Bayesian methods, support vector machines (SVMs), k-nearest neighbors, and neural networks (17).

Supervised learning has demonstrated particular value in exercise physiology, enabling structured prediction and categorization of physiological data, particularly in classification and regression tasks (18).

### Unsupervised learning

Unsupervised learning methods operate independently to identify patterns without human guidance. They work with unlabeled data to discover patterns, detect anomalies, identify frequently occurring items in a dataset (association rule mining), and reduce the number of variables in a dataset (dimensionality reduction) (19).

### Semi-Supervised learning

Semi-supervised learning combines labeled and unlabeled data, improving model accuracy when labeling data is costly or time-consuming (20). This approach balances the robust performance of supervised learning with the efficiency of unsupervised learning. The labeled data helps the algorithm learn relationships between data points, their characteristics, and their corresponding labels, which can then be applied to classify new unlabeled data. While labeled data generally improves algorithm performance, acquiring it can be expensive and time-consuming.

### Reinforcement learning

Reinforcement learning is an adaptive approach where algorithms learn by interacting with their environment, optimizing actions over time, and providing real-time feedback (21). This approach finds primary applications in robotics and video games. While less common in exercise physiology, this method has potential for future applications in providing adaptive training protocols.

## Applications in exercise endurance physiology

Machine learning has significantly advanced exercise physiology, particularly in endurance sports, by providing innovative methods to analyze complex physiological data and enhance endurance performance. The applications of ML techniques in this domain can be categorized into different areas.

### VO_2_max prediction

Accurate estimation of VO_2_max is crucial for assessing aerobic fitness and designing endurance training programs. Traditional methods require laboratory tools, like metabolic carts, and involve exhaustive exercise protocols that can be invasive, time-consuming, and impractical for large-scale or field-based assessments (14). By leveraging ML algorithms, researchers have developed models that predict VO_2_max using data from submaximal or non-exercise tests, enabling more accessible and frequent assessments.

Several supervised ML models have been developed for VO_2_max prediction, including multiple linear regression, SVMs, artificial neural networks (ANNs), and multilayer perceptrons (15). These models utilize inputs such as heart rate (HR), age, sex, body composition, and physical activity levels to estimate VO_2_max without the need for maximal exercise testing. Among these techniques, ANNs have often outperformed others due to their ability to capture complex nonlinear relationships between variables (15).

Advancements in ML approaches have further improved non-exercise VO_2_max prediction algorithms. Liu et al. (22) employed a Light Gradient Boosting Machine (LightGBM, supervised ML) on data from U.S. national surveys, achieving significantly better accuracy compared to existing non-exercise algorithms. Their model reduced the error by 12%–15%, demonstrating the potential of ML to enhance the generalizability and predictive power of VO_2_max estimation methods.

Beyond VO_2_max prediction from non-exercise data, ML models have been applied to predict VO_2_ responses during various physical activities. Beltrame et al. (14) utilized ML analysis of wearable sensor data to predict oxygen uptake dynamics during daily activities. Their regression model, trained on accelerometer and HR data, achieved high accuracy, facilitating continuous monitoring of aerobic metabolism in free-living conditions. This approach allows for real-time feedback and personalized exercise prescriptions based on individual metabolic responses.

Similarly, Borror et al. (23) predicted VO_2_ responses during cycling at varied intensities with ANNs. Using inputs such as HR, cadence, power output, and cycling speed, their model provided accurate estimations of VO_2_. The incorporation of mechanical and physiological data enhanced the model's predictive capability, highlighting the importance of combining diverse data sources in ML models.

Hedge et al. (24) employed a temporal convolutional neural network to predict VO_2_ kinetics during heavy intensity cycling exercise. By analyzing data from wearable sensors measuring HR, ventilation (VE), and breathing frequency (BF), they developed a model capable of providing real-time predictions of VO_2_ dynamics. This advancement is particularly valuable for high-performance athletes and clinical populations, where understanding VO_2_ kinetics can inform training adaptations and monitor rehabilitation progress.

Khurshid et al. (25) demonstrated the use of deep learning, a subset of ML that utilizes neural networks with multiple layers to model complex patterns in data, to predict VO_2_max from resting 12-lead ECGs. Their model analyzed ECG features to estimate VO_2_max without requiring exercise testing, achieving a concordance correlation coefficient of 0.80. This non-invasive and efficient method for assessing cardiovascular fitness can be applied in large-scale screenings and in populations where exercise testing is contraindicated.

### Estimation of physiological thresholds

Physiological thresholds like ventilatory thresholds (VT1 and VT2) are critical indicators of endurance performance. Traditionally, determining these thresholds requires exhaustive CPET and at least two physiologists (26). Metabolic indicators of endurance performance, as lactate thresholds (LT), need invasive procedures like blood lactate sampling, which can be impractical in many settings (6, 27, 28). Non-invasive alternatives by analyzing readily obtainable physiological signals are offered with ML.

Including recurrent neural networks (RNNs) and convolutional neural networks (CNNs), ML techniques have shown promising results in detecting ventilatory thresholds during CPET. Zignoli et al. (27) successfully applied these models to automatically detect VT1 and VT2 from CPET data, achieving expert-level performance. The models uncovered complex nonlinear relationships and demonstrated high competence in classifying exercise intensity levels.

Training algorithms on crowd-sourced CPET data has outperformed experts in finding ventilatory thresholds (6, 27, 29). However, neural network performance in detecting VT1 may deteriorate for individuals with poor aerobic fitness, indicating a need for more diverse training data. Incorporating these AI-assisted methods into CPET hardware and software could provide more objective and efficient analysis of exercise data.

Badawi et al. (30) reported success in estimating lactate thresholds using ML models trained on non-invasive parameters such as HR, perceived exertion, and power output. Their approach simplifies performance testing by removing the need for blood lactate sampling, allowing for more frequent monitoring of training adaptations.

Cho et al. (31) employed deep learning to estimate dynamic ventilatory thresholds from ECG data alone. By extracting features from ECG signals, their model could determine thresholds without additional respiratory measurements. This method simplifies the assessment process and enables continuous monitoring during training sessions, providing valuable feedback for adjusting exercise intensity in real time.

### Cardiovascular assessments and heart rate variability analysis

Cardiovascular health is paramount in exercise physiology, especially in endurance exercise (32). Machine learning has been employed to predict cardiovascular events during exercise testing. Shen et al. (33) compared several algorithms and found that Extreme Gradient Boosting (XGBoost, supervised ML) was most effective. This application underscores how ML can improve risk assessment and safety in cardiopulmonary exercise testing.

A crucial indicator of autonomic nervous system activity and the overall physiological state of endurance athletes is heart rate variability (HRV) (32). Advanced ML models, such as deep learning algorithms, have been developed to analyze HRV data, surpassing traditional methods. Hernández-Ruiz et al. (34) utilized support vector machines (SVMs) to classify HRV data, achieving an accuracy of 90.3% in determining whether HRV was decreased or increased. Ahmad et al. (35) applied artificial neural networks (ANNs) to predict levels of physical fatigue using HRV features in time and frequency domains. The model achieved an accuracy of 80.6% in classifying fatigue levels. Xu et al. (36) demonstrated the use of a deep recurrent neural network to extract pulse rate variability from photoplethysmography signals during intense physical exercise, effectively handling motion artifacts common in such signals.

### Prediction of exercise-induced hypoxemia a posteriori

Around 70% of endurance-trained athletes exhibit EIH (37). A simple diagnostic criterion is a drop in SpO_2_ of at least 4% between rest and maximal exercise during a CPET (7). Although oximetry is simple to use, EIH is not always measured, despite its known influence on specific adaptations to exercise in normoxia and hypoxia (38).

**Figure 1:**
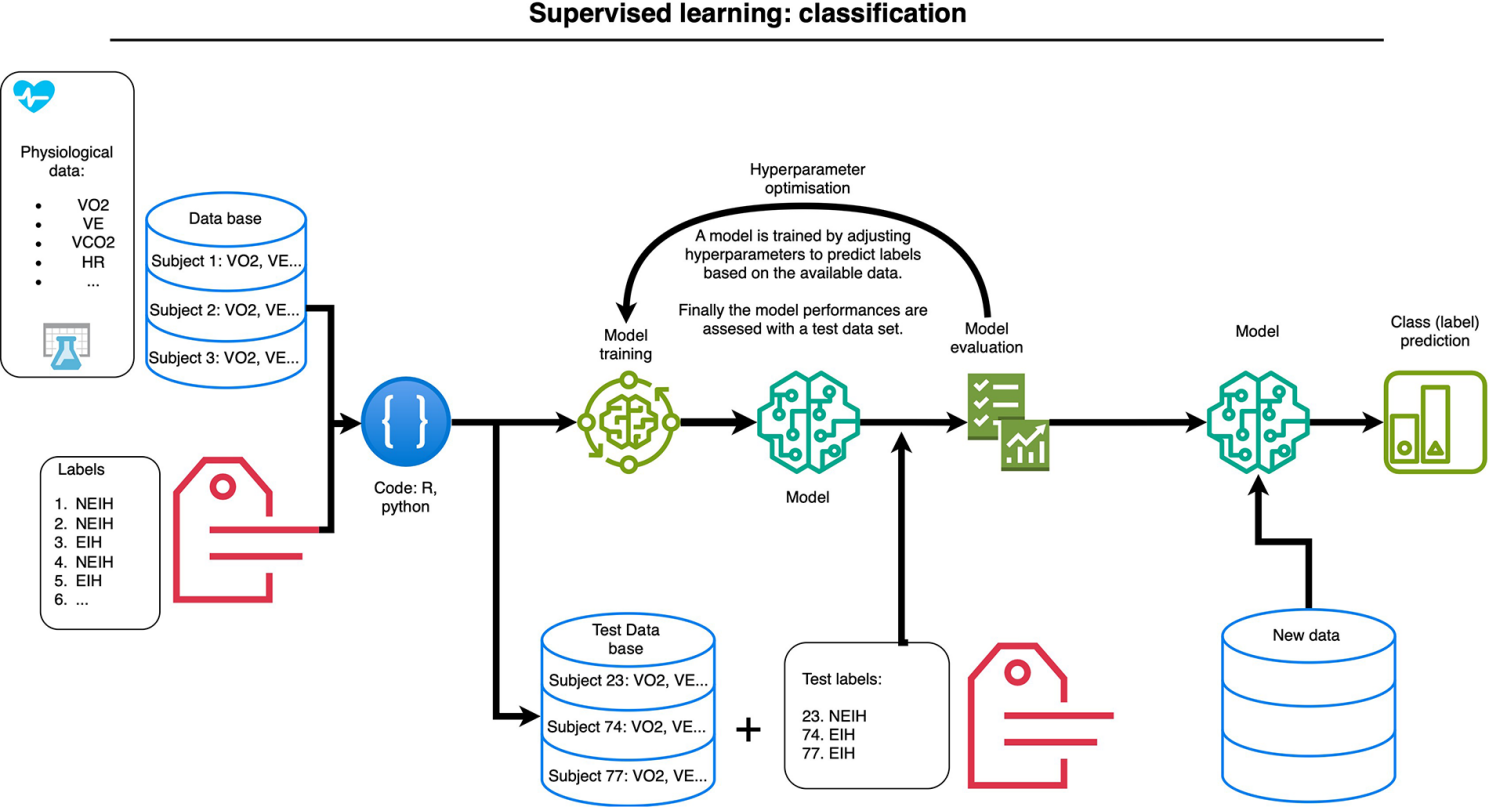
Supervised learning workflow used to predict exercise-induced hypoxemia (EIH) in endurance-trained athletes, without oxygen saturation measurements, based on previous cardiopulmonary exercise test parameters (not all included): oxygen consumption (VO_2_), ventilation (VE), carbon dioxide production (VCO_2_), heart rate (HR). The machine learning classifies athletes as either having or not having EIH [adapted from Boudry et al. (39)].

Supervised learning models, such as those used by Boudry et al. (39), have successfully predicted EIH based on parameters measured during a CPET without SpO_2_ measurement. Figure 1 shows the supervised learning workflow used to predict EIH in endurance-trained athletes without SpO_2_ measurement, using cardiorespiratory parameters of a CPET. These included ventilation (VE), oxygen consumption (VO_2_), carbon dioxide production (VCO_2_), HR, and ratios like VE/VCO_2_, VCO_2_/VO_2_, VE/VO_2_, and VO_2_/HR, in addition to demographic information. The data were then analyzed, and the results refined using labeled data indicating EIH or non-EIH (NEIH). This means that EIH or NEIH status can be determined a posteriori using previous data. This possibility could change the way we understand endurance adaptations, particularly in hypoxia, improving our understanding of exercise physiology.

## Strengths of machine learning in exercise physiology

One of the primary strengths of ML is its ability to handle large, high-dimensional datasets. ML algorithms can uncover complex, nonlinear relationships among variables that traditional statistical methods may overlook (40, 41). This capability is particularly valuable in analyzing physiological data, which often involves multiple interconnected variables.

The capacity to process real-time data from wearable sensors is another significant advantage. Wearable devices continuously collect high-frequency data on variables such as HR and VO_2_. Machine learning models can analyze this data instantaneously, providing immediate feedback that enables dynamic adjustments to training plans. For example, Gao et al. (13) demonstrated how real-time analysis can enhance performance monitoring.

By integrating diverse data types, machine learning facilitates the analysis of individual responses to exercise, accounting for the unique physiological profile of each athlete (42).

## Limitations of machine learning in exercise physiology

Machine learning in exercise physiology faces several key challenges, notably the risk of overfitting when small or homogeneous datasets are used, which restricts the generalizability across diverse populations. For instance, models trained to predict VO_2_max based on a specific population of elite athletes may not generalize well to recreational athletes or individuals with different physiological characteristics (15). Techniques like cross-validation and regularization help mitigate this issue by improving generalization (43). Additionally, gathering larger datasets that encompass a wide range of physiological profiles enhances the model's ability to generalize.

Model transparency is another concern, as deep learning often functions as a “black box,” obscuring the physiological basis for predictions (44). For instance, a deep learning model might accurately predict lactate thresholds but provides little insight into which physiological variables are most influential (27). This lack of transparency can reduce trust in ML applications, especially in critical decision-making contexts. Methods from Explainable Artificial Intelligence (XAI), can address this by providing interpretable insights into model outputs (45).

Lastly, there is often a trade-off between model complexity and interpretability, with simpler models, such as linear regression, offering clearer insights but at the expense of predictive accuracy (46). Balancing these factors is critical for effective ML applications in this domain.

## Challenges and future directions

### Interpretability

**Figure 2:**
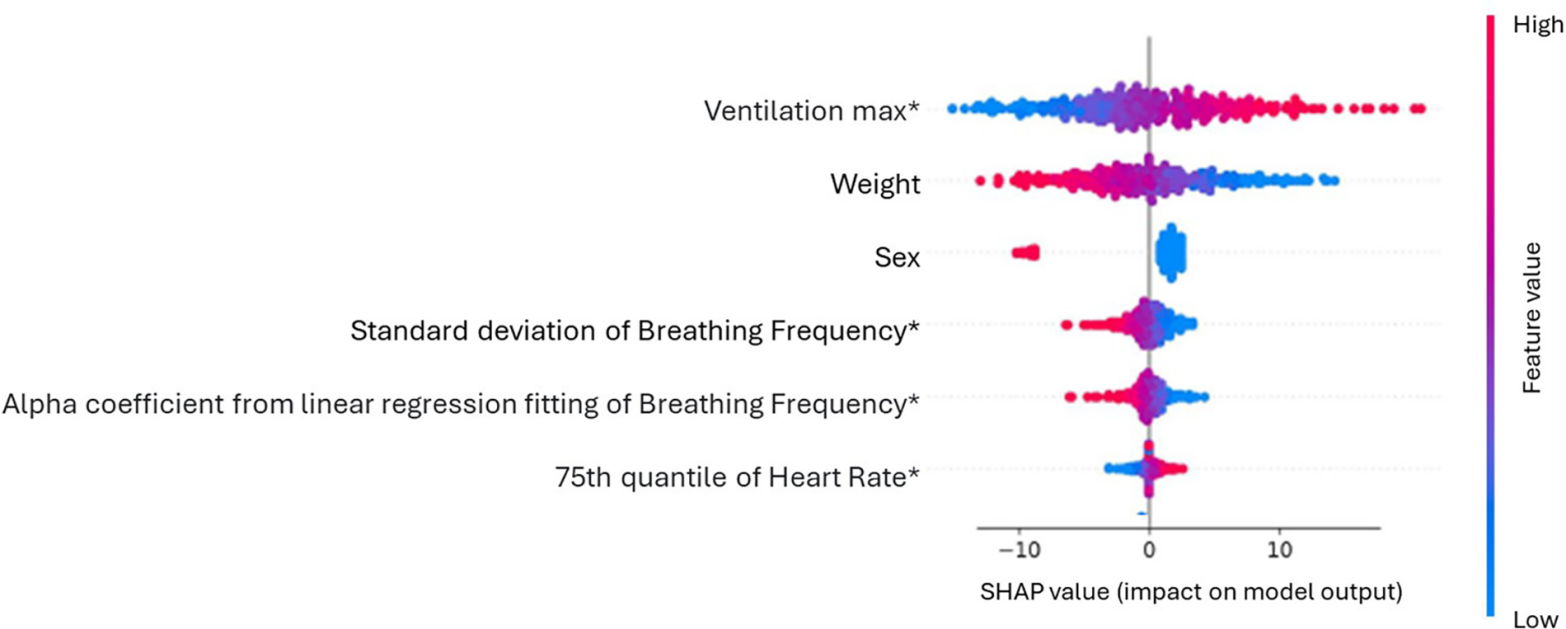
SHAP summary plot illustrating feature importance and effects in predicting maximal oxygen consumption (VO_2_max) based on submaximal cardiopulmonary exercise test (CPET) data [adapted from Rosoł et al. (51)].

The main challenge of ML in exercise physiology, as well as in other domains, is model interpretability, which allows practitioners to understand and trust outputs, facilitating informed decision-making (44). To address this issue, the field of XAI offers tools designed to enhance the transparency of ML models (47). Tools like ELI5 (48), LIME (49) and SHAP aid users in understanding the decision-making processes of ML models by visualizing the importance of individual features. For instance, SHAP values quantify the contribution of each feature to a prediction, offering insights into the model's workings (50). In studies using ML to analyze physiological data, SHAP summary plots have demonstrated the importance and effects of top features, helping exercise physiologists validate model accuracy. Figure 2 depicts a SHAP summary plot from a study by Rosol et al. (51) investigating the prediction of VO_2_max using ML techniques from demographic and cardiorespiratory parameters obtained during a submaximal treadmill CPET. In this example, a high maximal VE measured during the test up to 85% of the maximum HR predicted by age increases the predicted VO_2_max. Similarly, the 75th quantile of HR measured under the same conditions contributes positively to the prediction but to a lesser extent. Understanding the importance of features and their physiological significance remains the domain of exercise physiologists. Their expertise allows for a deeper interpretation of the model, ensuring that predictions align with known physiological principles. Selecting appropriate XAI tools is critical for ensuring clear communication of results, particularly when translating complex ML predictions into actionable insights for practitioners (52). If we take the example of EIH prediction, the next step will be to develop a tool capable of capturing CPET results to classify athletes as EIH or NEIH.

### Data quality

Enhancing data quality and availability is also essential. Standardizing data collection protocols and compiling high-quality datasets from diverse populations will improve ML model reliability and generalizability (53). Collaborative data-sharing initiatives can expand access to valuable datasets. Adopting technologies like federated learning can address privacy concerns by enabling ML training on decentralized data without sharing sensitive information (54), fostering collaborative research while protecting privacy.

### Ethical considerations

Another important aspect concerns ethical approaches. Ensuring that machine learning models are developed and deployed responsibly is crucial, particularly when dealing with sensitive physiological data. Privacy and data protection are paramount; researchers must adhere to ethical guidelines and regulations such as the General Data Protection Regulation (GDPR) to safeguard personal health information (55). Informed consent and transparency about data usage are essential to maintain participant trust. Additionally, ML models should be scrutinized for potential biases that could lead to unfair outcomes or misinterpretations, especially across different demographic groups (56). Ethical deployment also involves accountability mechanisms to address errors or unintended consequences. By embedding ethical considerations into the design and implementation of ML models, practitioners can ensure that advancements in exercise physiology benefit all stakeholders responsibly and equitably.

## Conclusion

The integration of machine learning into endurance exercise physiology is essential to save time for athletes and physiologists who must analyze data from both laboratory practices (e.g., CPET) and field settings (e.g., connected devices). However, exercise physiologists must strike a balance between model complexity and the quality of available data, as simpler models may often suffice and provide more interpretable results. By focusing on interpretability, data quality, and ethical approaches, the field can fully realize ML's benefits. To ensure that ML applications develop under optimal conditions and meet the challenges in the field, at the service of athletes and trainers, it is imperative to foster interdisciplinary collaboration between exercise physiologists, data scientists, engineers, ethicists and other stakeholders.
